# Ethical leadership and nurses’ job performance: the mediating role of self-compassion

**DOI:** 10.3389/fpubh.2024.1535065

**Published:** 2025-01-08

**Authors:** Xingxing Liu, Fang He, Tian Tian, Fangli Guo, Jun Zhang, Yuexia Zhong

**Affiliations:** Outpatient Department, The Second Affiliated Hospital of Air Force Military Medical University, Xi'an, China

**Keywords:** self-compassion, ethical leadership, nurses, job performance, the mediating role

## Abstract

**Background:**

Ethical leadership is crucial in nursing management, and self-compassion is increasingly recognized as a significant factor influencing nurses’ job performance. Although the link between ethical leadership and nurse job performance has been established, the specific mechanisms that underlie this relationship remain unclear. Additionally, there is a paucity of research examining the potential role of self-compassion in this context. This study aims to investigate the relationship between self-compassion, ethical leadership, and nurses’ job performance while also validating the mediating role of self-compassion.

**Methods:**

This study employed a convenience sampling method to conduct a cross-sectional online survey involving 968 nurses from four tertiary-level hospitals in Xi’an, China, conducted between April and May 2024. Participants completed self-report questionnaires that included the Ethical Leadership Scale, Self-Compassion Scale, and Job Performance Scale. Data analysis was performed using descriptive statistics, the Mann–Whitney U test, the Kruskal-Wallis H rank sum test, Spearman correlation analysis, and the PROCESS tool within SPSS.

**Results:**

The analysis revealed a significant positive correlation between ethical leadership and self-compassion (*r* = 0.631, *p* < 0.01), as well as between ethical leadership and job performance (*r* = 0.688, *p* < 0.01). Additionally, a positive correlation was found between self-compassion and job performance (*r* = 0.564, *p* < 0.01). Mediation analysis indicated that self-compassion partially mediated the relationship between ethical leadership and job performance. The overall impact of ethical leadership on job performance (*β* = 0.680) comprised a direct effect (*β* = 0.499) and an indirect effect mediated by self-compassion (*β* = 0.181). The mediating effect accounted for 26.62% of the total effect.

**Conclusion:**

The results of this study indicate that ethical leadership not only directly predicts nurses’ job performance but also indirectly influences it by enhancing their levels of self-compassion. Consequently, nursing managers should prioritize the cultivation and reinforcement of ethical leadership styles while fostering a supportive environment that promotes effective self-compassion practices. Implementing these strategies is essential for improving nurses’ job performance and well-being, ultimately contributing to a higher quality of care and greater stability within the nursing team.

## 1 Introduction

The current medical environment signifies a profound transformation and functional expansion of the role of nurses. Nurses have evolved from being mere technical operators within the traditional framework to becoming an indispensable core component of the modern social medical service system (1). This transformation is evident not only in the technical aspects of nursing practice but also in the comprehensive involvement of nursing staff in disease prevention, health education, patient care, and medical team collaboration, thereby underscoring their crucial role in enhancing the quality of medical services and patient experiences (2). While we acknowledge the indispensable value of the nursing industry, it is also essential to confront the current challenges and difficulties faced by this sector. The nursing industry experiences a high turnover rate, which is regarded as a significant contributor to nurse shortages (3). The “Global Status of Nurses Report,” published by the World Health Organization (WHO) in 2020, projects that the global nursing shortage will approach nearly 6 million individuals by 2030 (1). The high-intensity work environment resulting from COVID−19 has the potential to contribute to professional burnout and emotional exhaustion among nursing professionals, which has, in turn, significantly elevated the turnover rate of nursing staff (4). The high turnover and frequent job changes among nursing staff have considerably increased the workload and psychological pressure on clinical nursing personnel. This situation has subsequently led to a decline in the quality of nursing services and poses a serious challenge to the stability and long-term sustainable development of the entire nursing system. In light of this context, we are compelled to re-evaluate the management and leadership models within the nursing industry to identify innovative strategies that effectively enhance nurses’ well-being, work performance, and, ultimately, the overall quality of service (5).

In recent years, ethical leadership, recognized as a positive leadership style, has garnered increasing attention from scholars (6). Ethical leadership represents a natural amalgamation of ethics and leadership principles. It primarily manifests in leaders establishing standardized and appropriate behavioral norms for their followers through their actions and interactions with subordinates. Concurrently, this leadership style fosters rational behavior among followers by encouraging two-way communication and collaborative decision-making (6). Exemplary ethical leadership is grounded in high moral standards and a strong sense of responsibility. Such leaders typically prioritize the long-term interests of their followers and the organization when making decisions and taking actions. Additionally, they are attentive to the welfare and career development of their subordinates, dedicating themselves to fostering a positive working environment that ensures their subordinates feel respected and recognized in their professional journeys (7). The complexity and challenges inherent in the nursing profession increase the likelihood of nurses encountering ethical issues in the workplace (8). Compared to other professions, nurses experience greater moral pressure when addressing patient health, engaging in ethical decision-making, and managing interpersonal relationships. As early as 1860, Florence Nightingale recognized the significance of ethical leadership within the nursing profession and proposed essential ethical principles that such leaders should embody, including fairness, neutrality, honesty, and respect (9). Since 2004, Storr has taken the lead in integrating ethical leadership theory into nursing research (10). Over the past decade, the number of research topics related to ethical leadership in nursing has significantly increased, indicating a growing trend. This development enhances our understanding of the positive impact of ethical leadership and its critical value within nursing organizations.

Ethical leadership is essential in nursing management, significantly impacting nurses, patients, and overall organizational outcomes (9). First, ethical leadership is pivotal in fostering a healthy professional environment. A qualitative interview study conducted among Iranian nurses indicated that ethical leadership substantially enhances nurses’ organizational identity (11). Consequently, nurses experience increased trust and solidarity, which contribute to a safer and more secure work environment. Furthermore, ethical leadership promotes a positive communication atmosphere by demonstrating understanding and empathy toward nurses. Second, ethical leadership influences nurses’ professional conduct. British scholar Mastracci (12) explored the relationship between ethical leadership and nurses’ organizational citizenship behavior. His research revealed that, even in work environments characterized by a lack of ethics and morals, ethical leadership can still serve a protective function for nurses’ organizational citizenship behavior. Third, ethical leadership contributes to the stability of nursing human resources. Previous research has shown that ethical leadership significantly reduces professional burnout (12) and emotional exhaustion (13) experienced by nurses while enhancing their emotional stability (14), subjective well-being (15), and psychological empowerment (16). This, in turn, effectively improves nurses’ career satisfaction and decreases their intention to leave. Fourth, ethical leadership is crucial for enhancing patient safety and improving the quality of care (17, 18). Finally, ethical leadership may enhance both individual and team effectiveness. Walumbwa (19) conducted a comprehensive exploration of ethical leadership’s effectiveness from a team-level perspective and found that it can improve team performance by fostering nurses’ awareness of their roles and encouraging constructive feedback among team members. However, recent results from an online survey indicate that there is no direct relationship between ethical leadership and job performance (20). Specifically, ethical leadership exerts an indirect effect on nurses’ work performance through the mediating influence of learning goal orientation and the moderating effect of colleagues’ support. Given that research on the correlation between ethical leadership and job performance is relatively limited and has yielded inconsistent conclusions, it is essential to further investigate this correlation among nurses.

Self-compassion refers to an individual’s understanding, acceptance, and caring attitude toward their own difficulties and failures (21). It comprises three opposing components: self-kindness versus self-judgment, common humanity versus isolation, and mindfulness versus over-identification (22). The key elements of self-compassion exist in a non-synergistic state and collectively form a dynamic system. Self-compassion is regarded as a valuable personal trait and resource associated with positive aspects of mental health, and it is notably malleable (23). Research indicates that individuals with elevated levels of self-compassion exhibit enhanced emotional regulation capabilities, which can serve as a buffer against negative emotions (24). Furthermore, high self-compassion is significantly associated with greater personal well-being (25), increased job satisfaction and performance (26), as well as an improved quality of professional life (25). Additional research suggests that self-compassion may enhance work performance by helping individuals safeguard their psychological resources and mitigate stress responses during challenging situations (27). Self-Determination Theory (SDT) posits that individuals with higher levels of self-compassion tend to exhibit greater intrinsic motivation, which, in turn, encourages them to exert more effort (28). Moreover, work engagement serves as a motivating factor that can lead to enhanced job performance and increased job satisfaction (29).

Currently, various leadership styles have been shown to be closely related to self-compassion, including authentic leadership (30), servant leadership (30), and transformational leadership (31). Ethical leadership occupies a unique and significant position among these styles. Recent research indicates that this leadership approach plays a crucial role in motivating followers to engage in positive behaviors and maintain their psychological well-being. Eisenbeiss (32) conducted a multi-source study involving leader-follower dyads, revealing a significant association between ethical leadership and increased effort from followers. Furthermore, the study found that ethical leadership fosters higher levels of moral emotions and greater mindfulness among followers. Similarly, a cross-sectional study of nurses conducted by Si (33) confirmed a significant relationship between ethical leadership and workplace mindfulness. Additional research indicates (34) that followers who embrace ethical leadership are more likely to exhibit compassionate behavior toward their colleagues. This study assessed compassion through dimensions of self-compassion, including mindfulness, shared humanity, and compassion itself. Consequently, it can be speculated that a correlation may exist between moral resilience and self-compassion. However, to date, no studies have specifically investigated the relationship between ethical leadership and nurse self-compassion. Therefore, it is imperative to further explore and validate this relationship within the nursing population.

Social exchange theory (SET) posits that individuals make behavioral choices based on considerations of costs and benefits during social interactions (35). Within an organizational context, the interaction between leaders and followers can be conceptualized as a social exchange process, wherein leaders’ actions significantly influence followers’ work attitudes and behaviors. In turn, employees reciprocate through their commitment and investment in the organization. When these interactions yield mutual benefits, both parties are more likely to sustain and enhance their relationship (35). The interrelationship among ethical leadership, self-compassion, and work performance can be effectively examined through the lens of SET. Under this framework, ethical leaders exhibit exceptionally high levels of empathetic interaction, ethical behavior, and a sense of responsibility, consistently prioritizing the long-term interests of both their employees and the organization when making decisions and taking actions. This leadership style places significant emphasis on the welfare and career development of employees. It is dedicated not only to fostering a positive working environment but also to ensuring that employees feel valued and treated with integrity and fairness. Employees with higher levels of self-compassion tend to exhibit greater sensitivity to their own behaviors and circumstances. When they perceive care and support from their leaders, they are likely to demonstrate more positive work attitudes and behaviors based on the principle of mutual benefit (36). Additionally, self-compassion can enable employees to manage work-related stress more effectively and diminish their inclination toward self-criticism, thereby enhancing their emotional regulation capabilities. This improvement facilitates smoother communication and collaboration with leaders. In an environment that promotes self-compassion, ethical leadership is better positioned to address employees’ psychological needs (37), further enhancing their overall well-being. In this reciprocal relationship, ethical leaders are required to provide sufficient resources and support to foster effective interactions and cooperation. Concurrently, employees are expected to reciprocate the leader’s support by demonstrating commitment and actively investing in the organization. Such exchange relationships are interdependent; if leaders fail to provide adequate resources and support, employee trust and commitment may decline, ultimately adversely affecting job performance.

Ethical leadership, self-compassion, and job performance establish a positive, reciprocal relationship characterized by the exchange of resources and support, with each factor promoting and influencing the others. This dynamic not only enhances employees’ mental health (37) but also contributes to a reduction in employee turnover (38). However, there exists a notable gap in the current research literature regarding the simultaneous examination of the interplay between ethical leadership, self-compassion, and job performance within the nursing population. In this context, the present study employed the framework of social exchange theory to thoroughly investigate the relationships among nurses’ perceptions of ethical leadership, self-compassion, and job performance, while also validating the mediating role of self-compassion in the relationship between ethical leadership and job performance. This research aids in constructing a process model that elucidates how ethical leadership influences nurses’ job performance and serves as a valuable reference for nursing managers seeking to enhance job performance, improve mental health, and elevate the quality of nursing services. Utilizing mediation analysis, this study assessed the relationships among ethical leadership, self-compassion, and job performance, proposing the following specific hypotheses:

*H1*: Ethical leadership is positively related to job performance.

*H2*: Self-compassion is positively related to job performance.

*H3*: Ethical leadership is positively related to self-compassion.

*H4*: Self-compassion mediates the relationship between ethical leadership and job performance.

## 2 Methods

### 2.1 Design and participants

Between April and May 2024, we conducted a cross-sectional survey of nurses across four comprehensive tertiary-level hospitals in Xi’an, China, employing convenience sampling. The inclusion criteria for this study were as follows: (1) Participants must possess a valid nurse practitioner qualification; (2) Participants must have a minimum of 1 year of clinical nursing experience; (3) Participants should understand the specific content of the study and provide consent to participate in the surveys. The exclusion criteria were defined as: (1) Nurses who were currently on vacation or undergoing further training were ineligible for participation; (2) Nurses interning in the investigation department were also excluded. According to the sample size estimation method for descriptive research recommended by Kendall (39), a sample size of 10 to 20 times the number of variables is suggested. Our study involved 17 variables, comprising 8 sociodemographic items and 9 dimensions across three scales. Consequently, the required sample size was calculated to range from 170 to 340 cases. To address potential under-response, this study further increased the original sample size by 20%, resulting in a final required sample size ranging from 204 to 408 cases.

### 2.2 Data collection

The researcher initially engaged with the nursing departments of each hospital, providing a comprehensive explanation of the study’s content and objectives. After obtaining consent from the nursing departments, the researcher appointed an investigator at each hospital and provided standardized instructions. Each investigator was responsible for distributing pre-designed online questionnaires to nurses who met the survey criteria and addressing any questions the nurses encountered during the completion process to ensure successful submission of the questionnaires. To maintain the integrity of the survey, it was stipulated that each IP address could submit a response only once, thereby preventing duplicate entries. Furthermore, to mitigate the impact of incomplete responses on data quality, the questionnaire was designed to allow submission only after all fields had been completed. Participants in this study remained anonymous, and informed consent was obtained from each participant, who also retained the right to withdraw from the survey at any time. A total of 989 questionnaires were collected, with 21 low-quality responses eliminated after screening. Ultimately, 968 questionnaires were effectively recovered, resulting in an effective recovery rate of 97.88%.

### 2.3 Measurements

#### 2.3.1 Demographic questionnaire

We independently developed a sociodemographic questionnaire that captures essential information about nurses, including gender, age, years of nursing service, educational level, professional title, marital status, average monthly income, and employment type.

#### 2.3.2 Self-compassion scale

Nurses’ levels of self-compassion were assessed using the Chinese version of the Self-Compassion Scale (SCS), originally developed by Neff (22) and subsequently translated and revised by Chen (40). The SCS encompasses six dimensions: self-kindness, self-judgment, common humanity, isolation, mindfulness, and over-identification. Each dimension consists of five, five, four, four, four, and four items, respectively, culminating in a total of 26 items. Each item is rated on a 5-point Likert scale, ranging from “strongly disagree” to “strongly agree,” with scores varying from 1 to 5. Notably, the dimensions of self-judgment, isolation, and over-identification utilize a reverse scoring method. Total SCS scores can range from 26 to 130, with higher scores indicating greater levels of self-compassion. The overall Cronbach’s *α* coefficient for the scale was 0.84, while the Cronbach’s α values for each dimension ranged from 0.51 to 0.70, indicating that the scale demonstrates good internal consistency.

#### 2.3.3 Ethical leadership scale

The ethical leadership scale was developed by Brown (6). This unidimensional scale consists of 10 items, each rated on a scale from 1 to 5, where 1 indicates ‘never’ and 5 indicates ‘always.’ Consequently, the total score can range from 10 to 50, with a higher score reflecting a greater perceived level of ethical leadership among nurses. Notably, the Cronbach’s *α* coefficient for this scale was 0.87, indicating good internal consistency. Due to the limited number of items, this scale is straightforward to implement during the measurement process, making it widely utilized in research related to ethical leadership and effectively validated in Asia (41).

#### 2.3.4 Job performance scale

The job performance scale was initially developed by Borman (42) and later translated and validated by Yu (43), resulting in the Chinese version of the scale. This version comprises two dimensions and a total of 11 items: task performance and relationship performance, with 5 items pertaining to task performance and 6 items related to relationship performance. Task performance evaluates work efficiency, work quality, and other factors directly associated with the job, while relationship performance assesses employees’ proactive assistance to colleagues in problem-solving and their management of interpersonal relationships (42). In this study, the items were evaluated using a 5-point Likert scale, with responses ranging from 1 (strongly disagree) to 5 (strongly agree). The total score for the Chinese version of the job performance scale ranges from 11 to 55, with higher scores indicating better job performance. The scale is categorized into three levels based on the average item scores: scores below 3 indicate low performance, scores above 4 indicate high performance, and scores between 3 and 4 represent medium performance. The overall Cronbach’s *α* coefficient for the scale was 0.92, while the coefficients for the two dimensions were 0.86 and 0.88, respectively.

### 2.4 Ethical considerations

This study adhered to the Declaration of Helsinki and its subsequent amendments, receiving approval from the Ethics Committee of the Second Affiliated Hospital of Air Force Medical University in Xi’an, China. All participating nurses provided informed consent and understood the purpose and content of the study. The homepage of the online questionnaire functioned as an informed consent form, and participants were informed of the principles of voluntariness, anonymity, and confidentiality throughout the survey process. Additionally, participants retained the right to withdraw at any time without incurring any loss of benefits.

### 2.5 Data analysis

This research conducted a statistical evaluation using SPSS version 25.0 and PROCESS Macro (version 3.3). As an initial step, a normality assessment was performed, which revealed that the dataset did not meet the criteria for a normal distribution. Consequently, the measurement data were expressed as medians with interquartile ranges, while categorical data were reported as counts and percentages. These descriptive statistics were employed to analyze the sociodemographic profiles of nurses alongside their scores in ethical leadership, self-compassion, and job performance. Subsequently, the Mann–Whitney *U* test and the Kruskal-Wallis H test were utilized to examine variations in job performance associated with the sociodemographic characteristics of nurses. The interrelationship among the three primary variables—ethical leadership, self-compassion, and job performance—was assessed using Spearman correlation coefficients. Finally, PROCESS Model 4 was applied to investigate the mediating effect of self-compassion on the relationship between ethical leadership and job performance while controlling for all statistically significant covariates identified in the sociodemographic assessment (44). Additionally, to evaluate the impact of ethical leadership on nurses’ job performance, this research calculated bias-corrected percentile bootstrap distributions with 95% confidence intervals, based on 5,000 bootstrap replications (44). All statistical analyses were two-tailed, and the significance threshold was set at *p* < 0.05 to determine the significance of the differences.

## 3 Results

### 3.1 Participants’ demographic characteristics and their distribution by job performance scores

This study included a total of 968 participants, with women comprising the majority at 928, representing approximately 95.87% of the total sample. The age distribution indicated that the predominant age group was between 30 and 40 years, with 450 individuals in this category, accounting for 46.49% of the total. Regarding years of nursing experience, the majority of participants had between 11 and 15 years of work experience, totaling 330 individuals, which constitutes 34.09% of the sample. In terms of educational background, a significant number of participants held a bachelor’s degree, totaling 867 individuals and accounting for 89.57%. Concerning professional titles, 54.44% of the participants held the title of nurse practitioner or lower. In terms of marital status, married participants represented 62.29% of the sample. Additionally, the monthly income distribution revealed that the highest income bracket was between 5,001 and 10,000 yuan, with 603 individuals, which also accounted for 62.29% of the total. Notably, more than half of the participants were employed under a contract system. Detailed sociodemographic information about the participants is presented in Table 1.

**Table 1:** Participants’ demographic characteristics and their distribution by job performance scores (*n* = 968).

Characteristics	*N* (%)	Work engagement		
		Median (P25, P75)	*Z/H*	*p*
Gender				
Male	40 (4.13)	53.50 (34.00, 55.00)	−1.001^a^	0.317
Female	928 (95.87)	37.00 (36.00, 40.00)		
Age (years)				
<30	345 (35.64)	50.00 (32.00, 53.00)	4.816^b^	0.090
30 ~ 40	450 (46.49)	47.00 (37.00, 55.00)		
>40	173 (17.87)	49.00 (37.00, 54.00)		
Length of service in nursing (years)				
≤5	276 (28.51)	46.00 (32.00, 53.00)	25.049^b^	<0.001
6 ~ 10	247 (25.52)	53.00 (37.00, 55.00)		
11 ~ 15	330 (34.09)	45.00 (45.00, 54.00)		
>15	115 (11.88)	54.00 (54.00, 54.00)		
Educational level				
College or below	55 (5.68)	51.00 (35.00, 55.00)	4.329^b^	0.115
Undergraduate	867 (89.57)	49.00 (37.00, 54.00)		
Postgraduate or above	46 (4.75)	54.00 (46.25, 54.00)		
Professional title				
The nurse	441 (45.56)	50.00 (32.00, 54.00)	5.630^b^	0.060
Nurse practitioner	324 (33.47)	50.00 (35.75, 53.00)		
Nurse-in-charge or above	203 (20.97)	45.00 (45.00, 55.00)		
Marital status				
Unmarried	365 (37.71)	51.00 (32.00, 54.00)	−1.329^a^	0.184
Married	603 (62.29)	49.00 (37.00, 55.00)		
Average monthly income (RMB)				
≤5,000	48 (4.96)	45.00 (32.00, 53.00)	4.808^b^	0.090
5,001 ~ 10,000	865 (89.36)	49.00 (37.00, 54.00)		
10,001 ~ 15,000	55 (5.68)	55.00 (31.00, 55.00)		
Employment type				
Officially on staff	59 (6.10)	43.00 (31.00, 54.00)	4.463^b^	0.107
Contract system	852 (88.02)	49.00 (37.00, 54.00)		
Personnel agent	57 (5.89)	45.00 (33.00, 55.00)		

^a Z, b H.^

### 3.2 Descriptive statistical analysis of variable scores

The median values for ethical leadership, self-compassion, and job performance were 41.00, 83.00, and 49.00, respectively. The results of the descriptive statistical analysis for these variables are presented in Table 2.

**Table 2:** Descriptive statistical analysis of variable scores (*n* = 968).

Variables	Items	Minimum	Maximum	Median (P25, P75)
Ethical leadership	10	19.00	49.00	41.00 (32.00, 42.00)
Self-compassion	26	63.00	115.00	83.00 (74.00, 92.00)
Self-kindness	5	4.00	24.00	17.00 (14.25, 19.00)
Self-judgment	5	5.00	23.00	17.00 (13.00, 18.00)
Common humanity	4	3.00	19.00	12.00 (11.00, 14.00)
Isolation	4	2.00	18.00	12.00 (10.00, 14.00)
Mindfulness	4	3.00	19.00	15.00 (12.00, 15.00)
Over-identification	4	6.00	18.00	12.00 (10.00, 14.00)
Job performance	11	16.00	55.00	49.00 (37.00, 54.00)
Task performance	5	5.00	25.00	22.00 (20.00, 25.00)
Relationship performance	6	9.00	30.00	27.00 (17.00, 30.00)

### 3.3 Correlations of the study variables

Table 3 presents the results of the correlation analysis investigating the relationships among ethical leadership, self-compassion, and job performance. The Spearman correlation analysis demonstrated a significant positive correlation between ethical leadership and self-compassion (*r* = 0.631, *p* < 0.01). Additionally, a significant positive correlation was identified between ethical leadership and job performance (*r* = 0.688, *p* < 0.01). Furthermore, a notable positive correlation was observed between self-compassion and job performance (*r* = 0.564, *p* < 0.01).

**Table 3:** Correlations of the study variables (*n* = 968).

Variables	1	2	3	4	5	6	7	8	9	10	11
1	1.000										
2	0.631^**^	1.000									
3	0.408^**^	0.740^**^	1.000								
4	0.474^**^	0.681^**^	0.184^**^	1.000							
5	0.237^**^	0.395^**^	0.687^**^	−0.046	1.000						
6	0.571^**^	0.630^**^	0.085^**^	0.747^**^	−0.309^**^	1.000					
7	0.430^**^	0.605^**^	0.796^**^	0.072^*^	0.601^**^	0.055	1.000				
8	0.462^**^	0.672^**^	0.147^**^	0.738^**^	−0.213^**^	0.745^**^	0.098^**^	1.000			
9	0.688^**^	0.564^**^	0.463^**^	0.303^**^	0.362^**^	0.268^**^	0.492^**^	0.328^**^	1.000		
10	0.672^**^	0.515^**^	0.336^**^	0.326^**^	0.254^**^	0.315^**^	0.434^**^	0.435^**^	0.914^**^	1.000	
11	0.690^**^	0.593^**^	0.497^**^	0.328^**^	0.351^**^	0.295^**^	0.458^**^	0.376^**^	0.958^**^	0.816^**^	1.000

*p < 0.05, **p < 0.01, 1, Ethical leadership; 2, Self-compassion; 3, Self-kindness; 4, Self-judgment; 5, Common humanity; 6, Isolation; 7, Mindfulness; 8, Over-identification; 9, Job performance; 10, Task performance; 11, Relationship performance.

### 3.4 Mediating effect of self-compassion between ethical leadership and job performance

This study employed the PROCESS macro (version 3.3) to conduct a bootstrapping analysis aimed at investigating the mediating role of self-compassion in the relationship between ethical leadership and work performance. Throughout the analysis, we carefully considered and controlled for confounding variables to enhance the reliability and validity of our research findings. Specifically, we utilized the Mann–Whitney U test and the Kruskal-Wallis H rank sum test to assess the impact of various sociodemographic variables on job performance. In our model, sociodemographic variables were treated as independent variables, while job performance was designated as the dependent variable. Additionally, we included the number of years of nursing service as a control variable to mitigate its potential influence on the research outcomes (see Tables 1, 4).

**Table 4:** The mediating model of self-compassion between ethical leadership and job performance (*n* = 968).

Outcome variable	Predictor variable	*R*	*R^2^*	*F* (*df*)	*β*	*t*
Job performance						
	Ethical leadership	0.683	0.467	422.305	0.680	28.357^***^
	Length of service in nursing				0.015	0.637
Self-compassion						
	Ethical leadership	0.626	0.392	311.493	0.588	22.954^***^
	Length of service in nursing				0.129	5.079^***^
Job performance						
	Ethical leadership	0.724	0.524	354.322	0.499	17.712^***^
	Self-compassion				0.308	10.812^***^
	Length of service in nursing				−0.025	−1.079

***p < 0.001.

Table 4 presents the results of the mediation effect tested using 5,000 bootstrap samples. The findings indicated that ethical leadership significantly predicts job performance. Notably, the direct predictive effect of ethical leadership on job performance remained significant even when self-compassion was accounted for. Further analysis revealed that both ethical leadership and self-compassion demonstrated substantial predictive power regarding job performance (*β* = 0.680, *p* < 0.001; β = 0.308, p < 0.001). It is important to note that in the model incorporating self-compassion, the impact of ethical leadership on job performance was diminished (β = 0.499, p < 0.001). Additionally, the study found that ethical leadership significantly predicts self-compassion (β = 0.588, p < 0.001). These findings suggest that self-compassion serves as a partial mediator in the relationship between ethical leadership and job performance.

**Figure 1:**
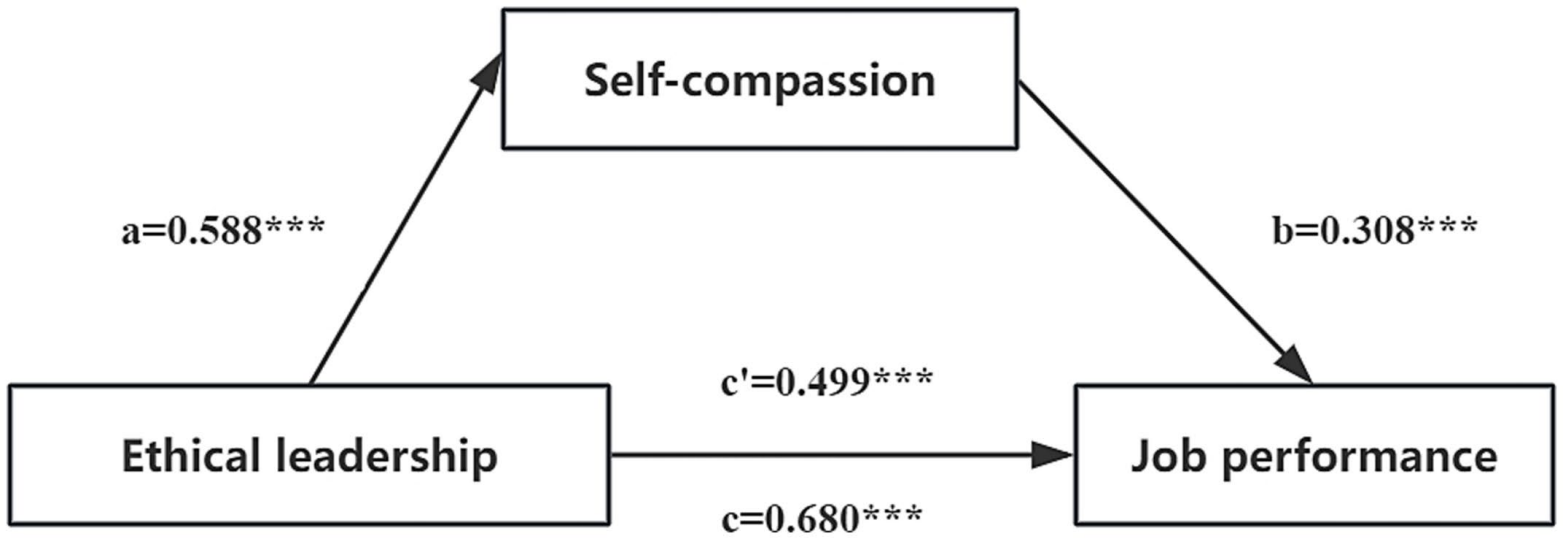
The mediating effect model of nurses’ self-compassion between ethical leadership and job performance. a, the effect of ethical leadership on self-compassion; b, the effect of self-compassion on job performance; c, the total effect of ethical leadership on job performance; c’, the direct effect of ethical leadership on job performance; ab, the mediating effect of self-compassion. ^***^p < 0.001.

The study revealed that the 95% bootstrap confidence intervals for the direct effect of ethical leadership on job performance, as well as the mediating effect of self-compassion, did not include zero (95% Boot CI = [0.444, 0.554]; [0.140, 0.225], see Table 5). This finding indicates that ethical leadership not only exerts a direct influence on job performance but also predicts job performance indirectly through the mediating variable of self-compassion. The relationships among the variables are illustrated in Figure 1. The direct effect was quantified at 0.499, while the mediation effect was measured at 0.181. Notably, the mediating effect accounted for 26.62% of the total effect, thereby highlighting the significant role that self-compassion plays in this process.

**Table 5:** Decomposition table of total effect, direct effect, and mediating effect (n = 968).

	Effect	Boot SE	95% Boot LLCI	95% Boot ULCI	The relative effect (%)
Total effect	0.680^***^	0.024	0.633	0.727	
Direct effect	0.499^***^	0.028	0.444	0.554	73.38
Mediating effect	0.181^***^	0.022	0.140	0.225	26.62

Boot SE, bootstrap standard error; 95% Boot LLCI, the lowest level of the 95% Bootstrap confidence interval; 95% Boot ULCI, the highest level of the 95% Bootstrap confidence interval.

***p < 0.001.

## 4 Discussion

The primary aim of this study was to investigate the relationship between ethical leadership and nurses’ job performance, while also analyzing the mediating role of self-compassion in this dynamic. The findings are expected to provide valuable insights for enhancing nurses’ job performance, bolstering their mental health, and ultimately improving the quality of care within healthcare settings. Firstly, we identified a significant positive correlation among ethical leadership, self-compassion, and job performance. Secondly, and most critically, the research confirmed that nurses’ self-compassion mediates the relationship between ethical leadership and job performance. These results suggest that when nurses perceive ethical support and care from their leaders, they are more inclined to exhibit self-compassion. This self-compassionate attitude helps alleviate the stress and challenges encountered at work, thereby enhancing work performance. The novelty of this study lies in its pioneering application of social exchange theory to conduct an in-depth examination of the interplay between ethical leadership, self-compassion, and job performance, as well as the mediating mechanism of nurses’ self-compassion. This research provides new insights for nursing managers, indicating that fostering a supportive and compassionate work environment can indirectly enhance both the performance and quality of care delivered by the entire team. Furthermore, these findings contribute to the development of targeted training and interventions aimed at promoting nurses’ self-compassion and overall professional well-being.

The scores of nurses’ assessments of ethical leadership were comparable to the findings of Qiu (45), yet significantly lower than those reported by McKenna (13) among Irish nurses. This discrepancy may be attributed to differences in years of experience. In this study, 54.03% of nurses had 10 years of working experience or less, a figure considerably higher than that in McKenna’s (13) research. Firstly, nurses with shorter tenure are often in the early stages of their careers, which may result in insufficient experience when evaluating and experiencing the role of leaders in ethical guidance. Secondly, these nurses may still be in the process of learning how to apply ethical theory to their daily practice, potentially leading them to underestimate the importance of ethical leadership in providing guidance and support. Consequently, even if leaders exhibit a high level of ethical leadership, nurses with less experience may not fully recognize their influence due to a lack of discernment and understanding (46). Therefore, hospitals and nursing schools could offer specialized ethics courses for less experienced nurses to help them establish a foundational framework for ethical judgment through systematic learning of ethical principles. This approach would enhance their understanding of the leaders’ roles in ethical decision-making. Additionally, fostering a positive ethical culture within the nursing team and establishing effective feedback and communication mechanisms can encourage less experienced nurses to express the ethical dilemmas they encounter in practice and share their perspectives on leadership in ethical decision-making, thereby improving their perceptions of ethical leadership.

The results of this study indicated that the SCS scores were at a moderate level, representing an improvement compared to the findings of Satake’s (47) survey among emergency department nurses; however, these scores remain lower than those reported in a cross-sectional study conducted in Italy by Özparlak (48). The specific reasons for this discrepancy may be attributed to differences in the working environment and the age distribution of the respondents. Notably, emergency department nurses often operate in high-pressure, fast-paced settings, where they must manage various emergencies, including patient trauma and death (47). This sustained exposure to high levels of stress and intense negative emotions may deplete nurses’ psychological resources, leading to burnout and compassion fatigue, which ultimately diminishes their capacity for self-compassion (49). Furthermore, in this study, only 17.87% of the nurses were over 40 years old, significantly lower than the 29% reported in Özparlak’s study (48). Typically, older nurses may exhibit higher levels of self-compassion due to their accumulated professional experience and personal growth. Therefore, nursing managers should recognize the critical importance of self-compassion for nurses’ mental health and strive to enhance workplace factors, such as rationally organizing work processes, flexibly allocating human resources, and providing adequate psychological support to mitigate nurses’ stress. Simultaneously, nurses should be encouraged to express and release negative emotions in a healthy manner to better confront challenges and adversity. Additionally, strengthening professional training related to self-compassion, particularly for younger nurses, will further enhance their levels of self-compassion.

The scores on the nurse job performance scale in this study were higher than those reported in a previous study conducted by Yu (50) in Henan, China. This discrepancy may be attributed to the differing years of nursing experience among participants in the two studies. In the current study, only 28.51% of nurses had less than 6 years of working experience, suggesting that the nurses in this study possessed greater clinical nursing expertise. Such nurses tend to demonstrate effective nurse–patient communication skills, a strong sense of responsibility, and career stability, all of which contribute to enhancing the overall job performance of the nursing team. Notably, variations in job performance scores among nurses in this study were observed based on years of nursing service, consistent with findings from prior research (50). Furthermore, both task performance and relationship performance are critical for improving nurses’ job performance. Task performance not only reflects the abilities exhibited by nurses in their work but is also directly linked to their work efficiency, serving as a comprehensive indicator of their professional skill proficiency (42). Enhanced task performance signifies an elevation in the overall nursing service level within the hospital and can lead to significant improvements in the hospital’s economic outcomes. Meanwhile, relationship performance is essential for fostering effective communication among various departments within the hospital. It contributes to cultivating a positive organizational communication atmosphere, thereby promoting overall job performance (42). Consequently, nursing managers should prioritize regular training to enhance nurses’ professional skills. Additionally, it is imperative to ensure the rational allocation of human resources and to provide adequate time and opportunities for knowledge exchange activities within the department to strengthen overall nursing performance.

The results of this study indicate that ethical leadership positively predicts nurses’ job performance, corroborating the findings of Walumbwa (19). Consequently, our study’s hypothesis 1 has been confirmed. A cross-sectional study by Qiu (45) demonstrated a significant correlation between ethical leadership, interactive justice climate, and organizational citizenship behavior. The interactive justice climate mediates the relationship between ethical leadership and organizational citizenship behavior, suggesting that ethical leadership may enhance nurses’ job performance through similar mediating factors, as organizational citizenship behavior is intrinsically linked to job performance. A systematic literature review (51) categorized the outcomes of ethical leadership and assessed the effect size, revealing an effect size of 0.45 for work or organizational outcomes. This finding indicates that ethical leadership is significantly associated with organizational-related outcomes; nurse job performance is a crucial component of overall organizational performance, thereby providing robust support for our research conclusions. McKenna’s research (13) noted that ethical leadership positively influences decision-making power and serves as a positive predictor of work engagement, while also being a negative predictor of emotional exhaustion and turnover intention. Both enhanced work engagement and reduced turnover intention contribute positively to job performance, further reinforcing our conclusion that ethical leadership is a positive predictor of nurses’ job performance. Additionally, while Zhang’s study (20) indicated no direct significant relationship between ethical leadership and job performance, it highlighted an indirect effect through learning goal orientation, with colleague support serving as a moderating factor. This finding deepens our understanding of the relationship between ethical leadership and job performance and indirectly corroborates the conclusions of this study. It is recommended that nursing organizations emphasize the importance of ethical leadership among nurse leaders and develop training programs based on the Ethical Leadership Scale. Furthermore, fostering an interactive and equitable environment within hospitals, along with establishing effective communication mechanisms, will enhance nurses’ job performance.

High levels of self-compassion may play a crucial role in helping nurses cope with compassion fatigue and burnout, thereby influencing their ability to manage stress and job performance (48). Research findings indicate a significant positive correlation between self-compassion and job performance, thereby supporting our hypothesis 2. Iranian scholar Bahrami (27) conducted a study on ICU nurses during the COVID−19 pandemic, revealing a positive correlation between self-compassion and job performance, with mental health serving as a mediating factor. This suggests that in high-stress nursing environments, self-compassion can foster a more favorable psychological state, enabling nurses to engage more effectively in their work and enhance their performance. A cross-sectional study by Özparlak (48) also identified a positive relationship between self-compassion levels and caring behaviors. Although this study did not directly assess job performance, caring behaviors are a critical component of it. Consequently, nurses with higher levels of self-compassion are likely to demonstrate greater patience and attentiveness in patient care, thereby improving care quality, which is a key indicator of job performance. Additionally, a study conducted among nurses in Henan Province, China, found that self-compassion was associated with work engagement, with negative emotions partially mediating this relationship (26). This implies that self-compassion may influence work engagement by modulating negative emotions, which subsequently impacts job performance, as work engagement significantly determines job performance. This further underscores the importance of valuing and fostering self-compassion among nurses to enhance overall job performance.

The findings of this study indicate that ethical leadership can enhance nurses’ levels of self-compassion, thereby supporting Hypothesis 3. Research by Eisenbeiss (32) suggests that ethical leadership is closely associated with followers’ moral emotions and mindfulness, implying that ethical leadership may foster self-compassion by influencing these emotional and cognitive states among nurses. As followers, nurses may activate their own moral emotions and enhance mindfulness through the active processing of moral information communicated by ethical leaders. These factors may be interrelated and contribute to self-compassion. Si (33) investigated the relationship between nurses’ well-being and ethical leadership, finding that workplace mindfulness serves as a partial mediator. Although the primary focus is on well-being, it can be inferred that the positive effects of ethical leadership on the workplace environment may similarly influence nurses’ self-compassion through analogous mechanisms. Specifically, a supportive leadership climate may extend its impact from the overall well-being of nurses to positively affect their self-compassion. A study conducted in the City of London found a positive correlation between ethical leadership and compassion, as well as peer-centered citizenship (34). This finding highlights the role of ethical leadership in fostering self-compassion among nurses. The moral influence exerted by leaders may encourage nurses to exhibit compassion toward their peers, which, in turn, could lead to increased self-compassion. It is recommended that nursing managers exemplify high moral standards and professional ethics while treating others with compassion and understanding, as their behaviors and decisions serve as role models for nurses to emulate. Moreover, prioritizing employee well-being is essential; this can be achieved by fostering open and honest communication, actively listening to nurses’ opinions and suggestions, and creating a supportive working environment where nurses feel valued and cared for. These strategies will help cultivate a culture of open communication, enabling nurses to alleviate work-related stress and improve their self-compassion.

The results of the mediation effect analysis revealed that nurses’ self-compassion significantly mediated the relationship between ethical leadership and job performance, accounting for 26.62% of the mediating effect. Consequently, our hypothesis 4 is fully supported. Our findings indicate that ethical leadership not only directly influences job performance but also indirectly enhances it by fostering nurses’ self-compassion. Firstly, ethical leaders demonstrate moral and ethical behavior patterns within the organization. They treat nurses fairly, respect their rights and interests, and show concern for their career development. This behavior represents a positive investment by ethical leaders in the social exchange relationship with nurses, who are then expected to reciprocate with positive work attitudes and efficient outputs. Secondly, nurses are able to perceive the positive behaviors exhibited by ethical leaders. According to SET (35), when one party provides positive resources or treatment to another, the latter is inclined to reciprocate. Nurses perceive their work environment as fair and respectful, fostering a psychological inclination to reciprocate. However, this reciprocity does not immediately translate into enhanced job performance; rather, it first influences the nurses’ level of self-compassion. Ultimately, the sustained positive actions of ethical leaders encourage nurses to maintain their self-compassion and improve their job performance, while the nurses’ enhanced performance further motivates ethical leaders to engage in positive behaviors. This dynamic creates a virtuous cycle that continuously enhances the operational efficiency of the entire organization. The research conducted by Si (33) supports the conclusions of this study. In his investigation, ethical leadership established a social exchange relationship in interactions with nurses. Ethical leaders engage in exchange relationships characterized by positive behaviors, such as treating nurses fairly and emphasizing career development. Nurses recognize these investments and demonstrate a willingness to reciprocate, a process influenced by workplace mindfulness. Mindfulness in the workplace serves as an internal resource; within a positive leadership environment, nurses are more likely to attain a state of mindfulness. For instance, when they feel respected by their leaders, they tend to be more focused on their work and experience a sense of tranquility, exemplifying workplace mindfulness. Consequently, this state positively impacts nurses’ happiness; when their emotional well-being is stable and affirmative, they derive greater satisfaction from their work. Additionally, Zhang‘s (20) research on the relationship between ethical leadership, learning goal orientation, colleague support, and nurses’ job performance further substantiates the conclusions of this study. The positive behaviors associated with ethical leadership represent an initial investment, anticipating high performance from nurses in return; however, this relationship is not direct. Learning goal orientation acts as a mediator, whereby the positive behaviors of ethical leadership serve as exchange signals for nurses. For example, ethical leadership encourages nurses to enhance their professional skills, fostering a learning goal orientation that prompts them to invest more effort in learning and improving their work to achieve success. This, in turn, generates positive rewards within the exchange relationship. Furthermore, colleague support plays a moderating role; a supportive colleague environment serves as an external resource that assists nurses in translating leadership influence into enhanced job performance.

## 5 Implication for nursing management

The most striking finding of our study is that self-compassion serves as a significant mediating factor between ethical leadership and nurses’ job performance. Our research provides novel insights into enhancing nurses’ job performance. Specifically, the mediating role of self-compassion within the relationship chain from ethical leadership to nurses’ job performance indicates that managers can ultimately improve the effectiveness of nursing services by fostering this quality. Furthermore, this study enhances the understanding of the interplay between leadership and nurses’ job performance, offering a new perspective for nursing management research. Nursing managers should recognize the critical role of self-compassion in enhancing nurses’ job performance and actively cultivate a work environment that promotes its development. This can be achieved by integrating more humanistic care into the management process and respecting the individual differences of nurses. Additionally, managers should prioritize the cultivation of an ethical leadership style, as it significantly influences nurses’ self-compassion and, consequently, their job performance. Furthermore, managers can implement relevant training or coaching initiatives to assist nurses in developing their self-compassion skills, thereby enhancing job performance and fostering a positive and efficient nursing team.

## 6 Limitations

Several limitations must be considered when interpreting the results of this study. This research employed a cross-sectional design, which, while facilitating data collection at a specific point in time, restricts the ability to verify causal relationships. To gain a deeper understanding of the dynamic interactions between ethical leadership, self-compassion, and nurse job performance, future research should consider utilizing a longitudinal design. This approach would enable the tracking of changes in these variables and their interactions over time. Furthermore, this study utilized convenience sampling, with participants selected exclusively from tertiary hospitals in the Xi’an City region, which somewhat limits the generalizability of the findings. To enhance the external validity of the results, it is recommended that future studies broaden the sample scope to encompass a wider range of hospital levels and geographical areas, thereby providing a more comprehensive reflection of the job performance of Chinese nurses and its influencing factors. Additionally, the data in this study primarily relied on self-reports from nurses, which may introduce expectation bias; that is, nurses might be influenced by social or personal expectations when reporting their job performance, potentially affecting the authenticity and accuracy of the results. Therefore, future research should consider employing multiple data collection methods, such as incorporating manager or peer evaluations, to enhance the credibility of the data and the validity of the research findings. In summary, this study is the first to investigate the mediating role of self-compassion between ethical leadership and nurses’ job performance, offering a novel perspective for improving both nurses’ job performance and mental health. Despite the limitations outlined in this study, our preliminary findings lay a foundation for future in-depth research. These findings highlight the positive influence of ethical leadership styles on nurses’ work and underscore the significance of cultivating self-compassion within nursing practice.

## 7 Conclusion

This study investigates the mediating role of self-compassion in the relationship between ethical leadership and nurses’ job performance, emphasizing the significant impact of ethical leadership styles on nurses’ professional outcomes. Research indicates that ethical leadership is a crucial factor influencing nurses’ job performance, as it enhances efficiency and service quality by fostering self-compassion among nurses. Self-compassion is vital in nursing management, enabling nurses to navigate stress and challenges in the workplace more effectively, which ultimately improves their overall job satisfaction and performance. In light of these findings, nursing managers should prioritize the development of an ethical leadership style and actively promote the enhancement of self-compassion among nurses. This approach not only contributes to improved job performance but also elevates the overall quality of nursing services. Furthermore, medical institutions must prioritize the mental health of nurses and cultivate a healthy, supportive working environment to facilitate the overall development and professional growth of nursing staff.

## Data Availability

The original contributions presented in the study are included in the article/Supplementary material, further inquiries can be directed to the corresponding author.
